# Fusion between cancer cells and macrophages occurs in a murine model of spontaneous *neu*^+^ breast cancer without increasing its metastatic potential

**DOI:** 10.18632/oncotarget.11508

**Published:** 2016-08-22

**Authors:** Michela Lizier, Achille Anselmo, Stefano Mantero, Francesca Ficara, Marianna Paulis, Paolo Vezzoni, Franco Lucchini, Giovanni Pacchiana

**Affiliations:** ^1^ Milan Unit, Istituto di Ricerca Genetica e Biomedica, CNR, Milan, Italy; ^2^ Humanitas Clinical and Research Center, Rozzano, Milan, Italy; ^3^ Centro Ricerche Biotecnologiche, Università Cattolica del Sacro Cuore, Cremona, Milan, Italy

**Keywords:** neu oncogene, cell fusion, metastatic spread, macrophage, reporter genes

## Abstract

Cell fusion between neoplastic and normal cells has been suggested to play a role in the acquisition of a malignant phenotype. Several studies have pointed to the macrophage as the normal partner in this fusion, suggesting that the fused cells could acquire new invasive properties and become able to disseminate to distant organs. However, this conclusion is mainly based on studies with transplantable cell lines. We tested the occurrence of cell fusion in the MMTV-neu model of mouse mammary carcinoma. In the first approach, we generated aggregation chimeras between GFP/neu and RFP/neu embryos. Tumor cells would display both fluorescent proteins only if cell fusion with normal cells occurred. In addition, if cell fusion conferred a growth/dissemination advantage, cells with both markers should be detectable in lung metastases at increased frequency. We confirmed that fused cells are present at low but consistent levels in primary neoplasms and that the macrophage is the normal partner in the fusion events. Similar results were obtained using a second approach in which bone marrow from mice carrying the Cre transgene was transplanted into MMTV-neu/LoxP-tdTomato transgenic animals, in which the Tomato gene is activated only in the presence of CRE recombinase. However, no fused cells were detected in lung metastases in either model. We conclude that fusion between macrophages and tumor cells does not confer a selective advantage in our spontaneous model of breast cancer, although these data do not rule out a possible role in models in which an inflammation environment is prominent.

## INTRODUCTION

Neoplastic transformation is a multistep process [1] although there could be many roads to the acquisition of the full spectrum of malignancy. In addition to oncogenes and tumor suppressors, a number of other genes including those implicated in migration, immune recognition, cell-cell interaction, have been associated to this process.

A distinct event which could potentially contribute to the acquisition of additional properties of the malignant cell is cell fusion. Cell fusion occurs in a restricted number of normal tissues, including placenta, muscles and osteoclasts [2]. One mechanism by which cell fusion could increase the malignant potential is polyploidy, which can predispose of its own to aneuploidy, since an abnormal content of DNA is unstable [3] (and references therein). The role of tetraploidy as an intermediate on the road to aneuploidy and cancer has directly been tested in p53-null mice [4]. In addition to challenging genome integrity, cell fusion with normal cells could provide the neoplastic cells with a full spectrum of gene expression programs which could be the target of selection by the environment, leading to the acquisition of a full malignancy status, including the ability to metastatize.

This possibility has been raised in the past [5–9]. More recently, several articles have been published dealing with the putative role of cell fusion in experimental cancers [10–18]. Surprisingly, although the last ten years have brought sophisticated animal models and powerful tools for analysis of cell fusion at the single cell level, to our knowledge, there is still no study performed in the context of spontaneous tumors occurring *in vivo*. All the reported studies start from *in vitro* cultured cell lines where fusion is obtained with cells of various origins, which are subsequently injected in immunocompromised or syngenic mice and evaluated for their malignant potential and/or acquired properties such as invasion and metastatization abilities. However, we feel that the artificial character of these studies and the selection occurring *in vitro* could not be representative of the normal development of malignancy in real tumors [19–22]. The choice of *in vivo* systems which are as similar as possible to the human situation is a fundamental requisite for translational studies in tumor biology [23].

In this paper we overcome these limitations by exploiting the MMVT-neu model which has been used by us and others to investigate both pathogenic issues and therapeutic aspects [20–22, 24]. In order to detect fusion between neoplastic and normal cells we developed two different approaches based on the MMTV-neu mouse which gave us the opportunity to study the presence of fused cell in a spontaneous tumor model.

## RESULTS

The approach initially used in our work is based on embryonic chimera production between a MMTV-neu (hereafter referred to as “neu”) mouse carrying a reporter gene and a normal mouse carrying a second reporter gene. To this aim, the two fluorescent GFP (Green Fluorescent Protein) or RFP (Red Fluorescent Protein) mice were individually crossed to the neu strain, in order to produce GFP/neu and RFP/neu double transgenic mice. Tumors arising in these mice will bear the color of the strain from which they are derived (data not shown).

**Figure 1:**
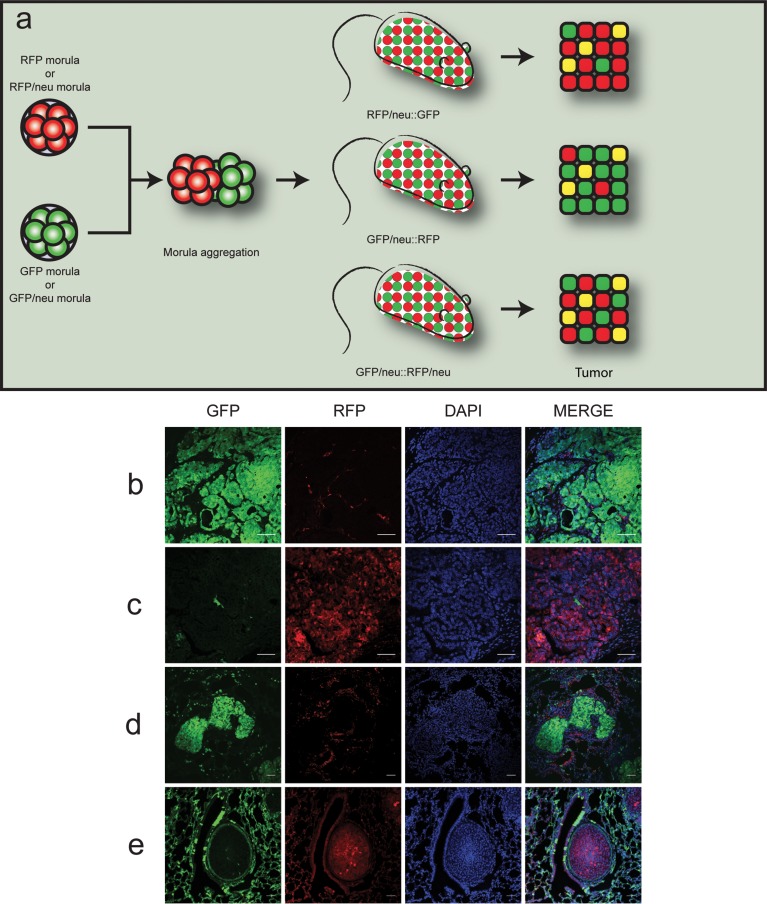
Chimeric double-fluorescent model for the study of cell fusion *in vivo* **a.** Outline of the morula aggregation approach. Morulae derived from RFP or RFP/neu morulae were aggregated with GFP or GFP/neu morulae, resulting in RFP/neu::GFP, GFP/neu::RFP or GFP/neu::RFP/neu animals. Tumors would thus be composed of either GFP (green) or RFP (red) expressing cells. Fused cells could be identified by co-expression of the fluorescent proteins (yellow color). (b-e) Representative confocal images of tumors and lung metastases obtained from chimeric animals (scale bars = 50 μm). **b.** Tumor in GFP/neu::RFP mouse; **c.** tumor in RFP/neu::GFP mouse; **d.** green metastasis in GFP/neu::RFP mouse; **e.** red metastasis in RFP/neu::GFP mouse. Cells nuclei were stained with DAPI in blue.

To analyze the occurrence of cell fusion, chimeric mice made by morula aggregation from the two double transgenic strains were produced. As schematically represented in Figure 1a, three pertinent types of chimeric mice can be generated: GFP::RFP/neu, which develop red tumors; GFP/neu::RFP, which develop green tumors; and GFP/neu::RFP/neu, which will develop both green and red tumors.

In order to investigate the putative fusion events between normal and neoplastic cells, we focused on GFP::RFP/neu and GFP/neu::RFP chimeric mice. Tumors developing in the breasts or salivary glands of these chimeric mice will appear green or red according to whether the transformed cell originated from the GFP^+^ or RFP^+^ component. Cells with both markers will be detected only if cell fusion occurred. Of note, not all the cells of individual GFP or RFP mice express the fluorescent protein, possibly in relation to transgene silencing randomly occurring in adult cells. This translates in the presence of a double negative population, thus underestimating the number of fusion events. Moreover, fusion between normal and neoplastic cells of the same genotype cannot be detected.

Nine chimeric mice were obtained. All females developed multiple mammary tumors, as expected, whereas male mice developed tumors originating from salivary glands. Therefore, the chimeric mice recapitulate the tumor development of our MMTV-neu mice previously described [19, 20, 24], even if part of their glands are composed by normal, neu-negative cells. This is in keeping with the high malignancy associated to *neu* oncogene overexpression. Histological analysis of these primary tumors identified the expansion of the neoplastic population showing either GFP or RFP, leaving in the mammary gland only a minor population of the reciprocal fluorescence (Figures 1b and 1c). Interestingly, metastases to the lung and their fluorescence were easily identified and evaluated (Figures 1d and 1e).

**Figure 2:**
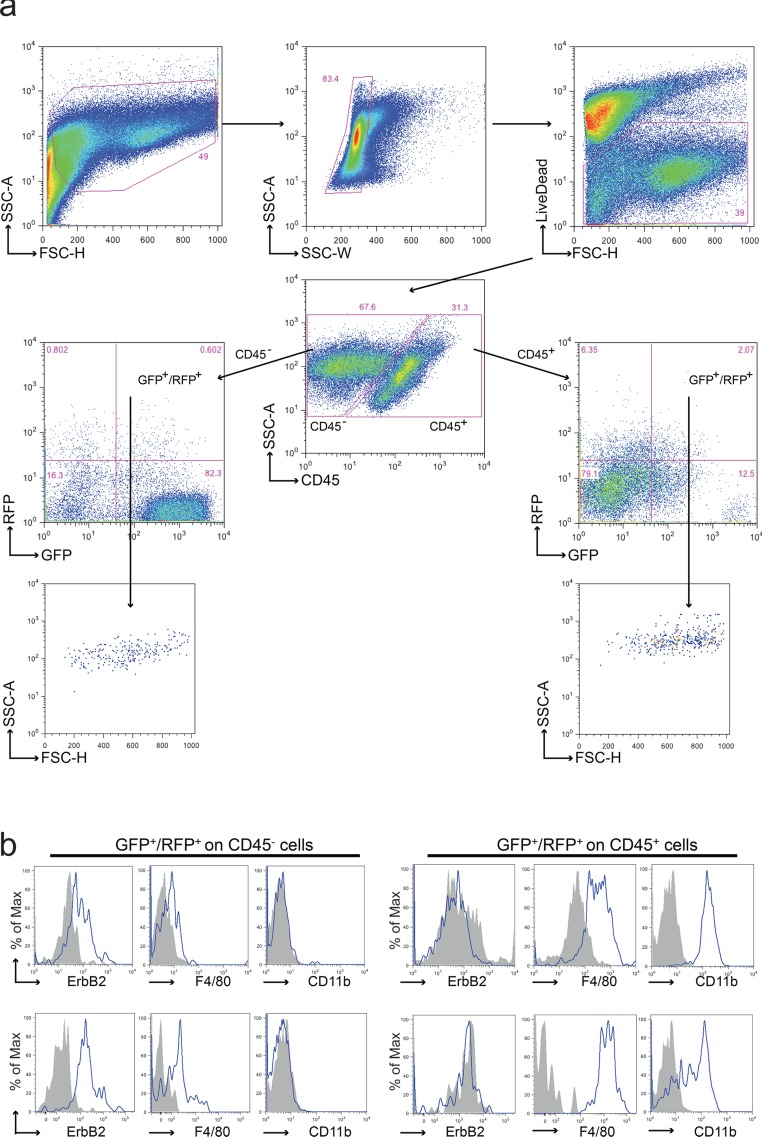
Analysis of cell fusion in double fluorescent animals **a.** Representative FACS analysis of a tumor derived from a GFP/neu::RFP chimeric animal. Upon doublets and death cells exclusion, leukocytes were discriminated from tumor and stromal cells using anti-CD45 antibody. Both CD45^−^ and CD45^+^ sub-populations were analyzed for the expression of GFP and RFP. GFP^+^/RFP^+^ cells were observed in both CD45^−^ and CD45^+^ sub-populations; these events were characterized by a well-defined morphology (high FSC and SSC values) supporting the absence of debris in the gated region. Each gated region was defined using the appropriate FMO negative control. **b.** Representative flow cytometric analysis of CD45^−^GFP^+^RFP^+^ and CD45^+^GFP^+^RFP^+^ sub-populations derived from two distinct tumors. The tumor marker ErbB2, the macrophage marker F4/80 and the myeloid marker CD11b were analyzed in both subpopulations. ErbB2 resulted expressed on CD45^−^GFP^+^RFP^+^ cells, CD11b just on CD45^+^GFP^+^RFP^+^ cells, while F4/80 was expressed in both subpopulations albeit at different levels. Grey fill histograms represent isotype controls plus Fluorescence Minus One (FMO), while blue lines are referred to ErbB2 or F4/80 or CD11b stained samples.

Cell populations obtained from primary tumors were analyzed by FACS. Live cells were examined for CD45 expression, a marker restricted to hematological cells and both CD45^+^ and CD45^−^ cells were investigated for the expression of the fluorescent markers. In Figure 2a, the analysis of a GFP^+^ tumor arising in a GFP/neu::RFP chimera is shown. While most cells displayed only GFP fluorescence, a small population showing both GFP and RFP was detected in both CD45^+^ and CD45^−^ populations.

Macrophages have been identified as fusion-prone cells in several systems. The double fluorescent cells were analyzed for the expression of ErbB2, the product of the *neu* oncogene which identifies neoplastic cells, and for F4/80 and CD11b, two markers of macrophages usually restricted to the CD45^+^ population. Most CD45^−^/RFP^+^/GFP^+^ cells displayed ErbB2 and F4/80 positivity but were negative for CD11b (Figure 2b), suggesting that fusion has occurred between tumor cells and macrophages with acquisition of only a subset of the genes expressed by macrophages. This partial acquisition is a frequent event in fusion between cells (see Discussion for further comments). On the contrary CD45^+^/RFP^+^/GFP^+^ cells expressed both F4/80 and CD11b markers but not ErbB2, suggesting that they might represent phagocytosis of neoplastic cells by macrophages or fusion between non-neoplastic cells, including intra-hematopoietic cell fusion.

**Figure 3:**
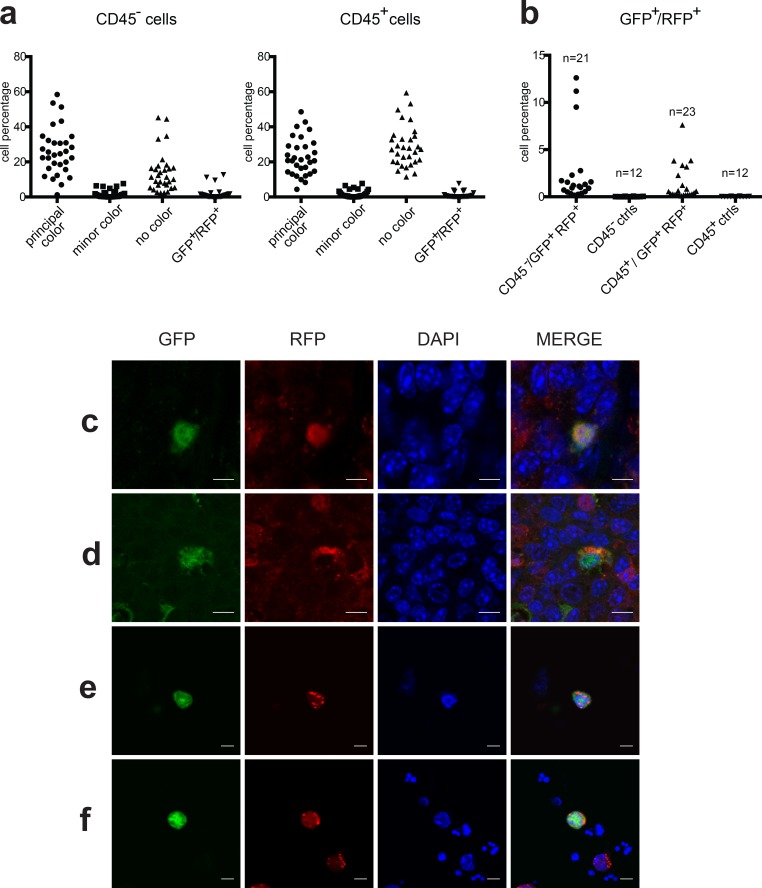
Analysis of double positive cells **a.** Percentage of CD45^−^ and CD45^+^ cells expressing fluorescent markers in each of the 31 tumors analyzed. Cells were classified as Principal color, Minor color, No color or GFP^+^/RFP^+^ color. **b.** Percentage of GFP^+^/RFP^+^ cells in CD45^−^ and CD45^+^ populations in 21 and 23 tumors respectively of double positive chimeric mice compared to that in twelve control tumors (9 single fluorescent and 3 non-fluorescent tumors). (c and d) Representative confocal images of GFP^+^/RFP^+^ tumor cells observed in FFPE chimeric tumor slices. (e and f) Representative confocal images of GFP^+^/RFP^+^ cells observed on cytospinned preparations obtained from tumor single cell suspensions. Cells nuclei were stained with DAPI in blue. Scale bars = 50 μm.

In total, 31 tumors from the 9 chimeric mice were analyzed by FACS for the presence of both GFP and RFP markers. Figure 3a summarizes the percentage of the various fluorescent live cell populations in each tumor according to the CD45 positivity. The chart shows the distribution of the four populations according to the fluorescent marker expression obtained by FACS analysis. The main population with a single marker (principal color) is in agreement with the tumor genotype; the minor single marker population (minor color) derives from the second genotype of the chimera; the “no color” population represents cells of either genotype which have shut-off marker expression; the double positive marker cells (GFP^+^/RFP^+^) represent the fused cell population in each tumor. We observed double positive GFP^+^/RFP^+^ cells (threshold value ≥ 0.1%) in 26 tumors out of 31 (83.8%). These 26 positive tumors were then analyzed for the distribution of double positive cells in the CD45 positive and negative fractions: 18 tumors displayed both CD45^+^ and CD45^−^ populations, 5 only CD45^+^ and 3 only CD45^−^. Figure 3b shows that the percentage of GFP^+^/RFP^+^ cells in tumors of chimeric mice ranged from 0.16% to 12.6% of live cells in the CD45^−^ population (mean = 2.50%, S.E.M. = ±0.80%) and from 0.1% to 7.6% in the CD45^+^ population (mean = 1.34%, S.E.M. = ±0.38%), whereas no double positive cells were detected in tumors of 12 control animals, including MMTV-neu mice expressing a single or no marker.

To further confirm the presence of GFP^+^/RFP^+^ fused cells, we looked for the presence of these rare events in histological sections derived from chimeric tumors. Examples of positive cells identified by confocal analysis on tumor slices are shown in Figures 3c and 3d, while double positive cells cytospinned from single cell tumor suspension are shown in Figures 3e and 3f.

In order to investigate *in vivo* whether cell fusion provides a greater ability to metastasize, we looked for the presence of double positive cells into lung metastases, which reproducibly occurs in most MMTV-neu mice [19, 20], by immunohistochemistry analysis. We reasoned that if a fused cell acquired greater proliferative ability and/or greater migration capability, this should translate in a detectable number of metastases bearing both GFP and RFP. However, this did not appear to be the case, since we were unable to detect any double positive cells in all the metastases examined from all chimeric mice.

These data strongly suggest that in our specific model cell fusion indeed occurred, although as a rare event. Our findings also identify the macrophage as one of the putative partners in the fusion between neoplastic and normal cells. To further investigate this point, we set up a second protocol in which the cell fusion can be identified only if occurred between the tumor cells and cells originating from the hematological compartment. In this approach, we exploited a different two-transgenic mouse system in which a Tomato reporter gene, which is not basally expressed due to the presence of a STOP cassette flanked by *lox*P sequences, is activated when exposed to the CRE recombinase. Double transgenic Tomato/MMTV-neu (Tomato/neu) animals were irradiated and transplanted with whole BM from the CRE strain (Supplementary Figure S1a): by this approach we generated Tomato/neu mice in which all the hematopoietic cells, including macrophages, express the CRE recombinase. In this way, if cell fusion between a neoplastic and a hematopoietic-derived normal cell occurs, the Cre transgene permanently activates the Tomato reporter protein which will be easily detected by cytofluorimetric or immunohistochemical techniques. This second approach, in addition to focusing on fusion between the hematological component and cancer cells, is also technically simpler since fused cells are identified based on a single fluorescent marker on a non-fluorescent background.

Transplants were performed as described in the Materials and Methods section, and the level of engraftment was evaluated by genomic real time PCR. Twelve Tomato/neu female mice were transplanted with BM from CRE male mice, while eight Tomato/neu males were transplanted with BM from female CRE mice. As shown in Supplementary Figures S1b and S1c, the protocol produced mice with an almost complete replacement of the donor hematological compartment. It must be considered that the Cre transgene maps to the X chromosome in our strain and, for this reason, animals transplanted with female BM are analyzed separately and compared to male CRE^+/−^ or female CRE^+/+^ mouse DNA as standard.

**Figure 4:**
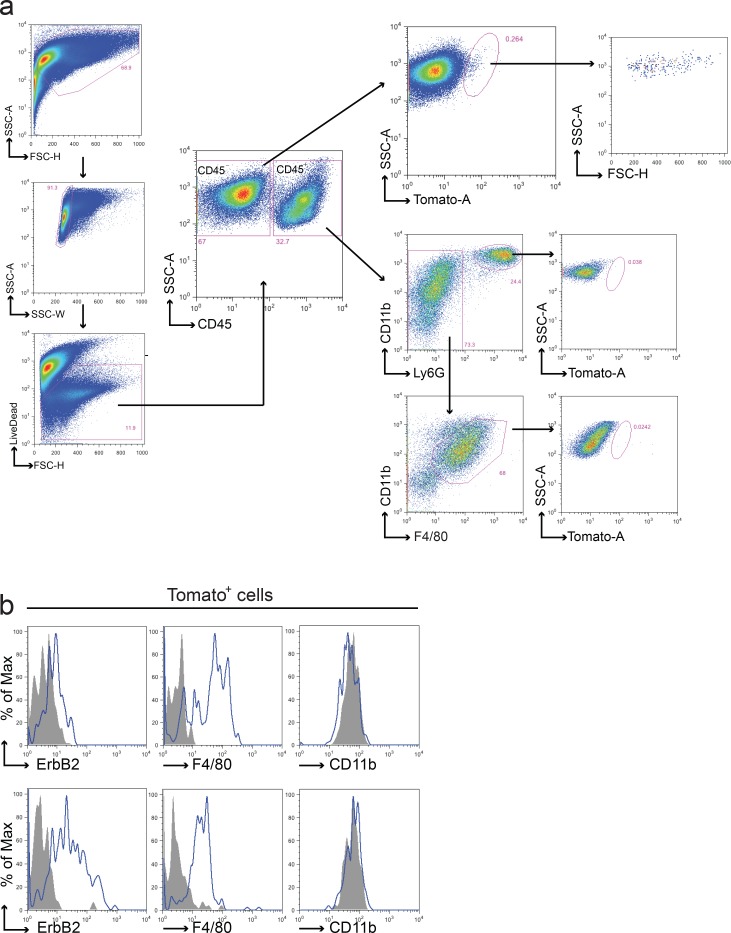
Fusion between cancer cells and macrophages in the BMT chimeric model **a.** Representative FACS analysis of tumors from Tomato/neu animals transplanted with CRE^+^ BM. Upon doublets and death cells exclusion, leukocytes were discriminated from tumor and stromal cells using anti-CD45 antibody. CD45^−^ and CD45^+^ live populations were analyzed for the presence of Tomato^+^ cells. Tomato^+^ cells were found in CD45^−^ population only and were characterized by a well-defined morphology (high FSC and SSC values) supporting the absence of debris in the gated region. Both macrophages and polymorphous nucleated cells presented in the CD45^+^ tumor population were negative for Tomato expression. Each gated region was defined using the appropriate FMO negative control. **b.** Representative flow cytometric analysis of ErbB2, F4/80 and CD11b on the Tomato^+^ sub-population derived from two distinct tumors. In agreement with the previous model, Tomato^+^ fused cells expressed ErbB2 and F4/80 on their surface but resulted negative for CD11b. Grey fill histograms represent isotype controls plus Fluorescence Minus One (FMO), while blue lines are ErbB2 or F4/80 or CD11b stained samples. Both macrophages and polymorphous nucleated cells presented in the CD45^+^ tumor population were negative for Tomato expression.

FACS analysis was performed on 45 primary tumors with a strategy similar to that applied to the GFP/RFP model (Figures 4a and 4b).

**Figure 5:**
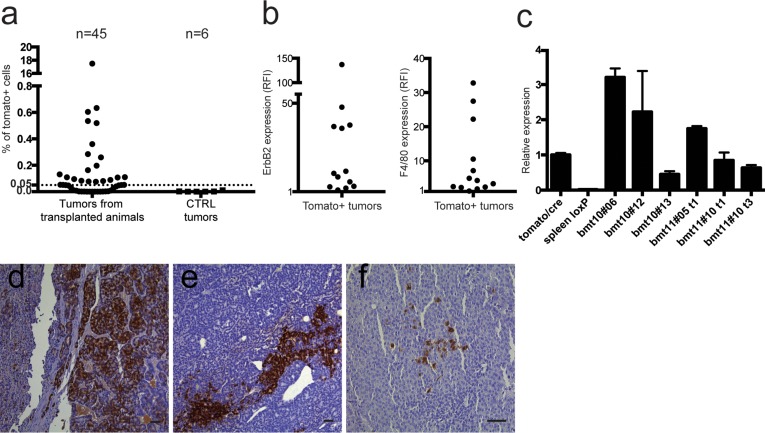
Analysis of Tomato positive cells **a.** 26 out of 45 (57.7%) tumors analyzed by flow cytometry were positive for Tomato expression, restricted to the CD45^−^ compartment, above an arbitrary threshold of 0.05% of live cells. None of the tumors of control animals (MMTV-neu and LoxP-tdTomato/neu mice) showed any Tomato^+^ cells. **b.** The left panel shows ErbB2 expression ratio between the CD45^−^/Tomato^+^ population and the same tumor population stained with isotypic control, expressed as Relative Fluorescence Intensity (RFI). The right panel shows F4/80 expression ratio between the CD45^−^/Tomato^+^ population and the same tumor population unstained for F4/80, expressed as RFI. Thirteen out of 26 tumors (50%) express both F4/80 and ErbB2 in the CD45^−^/Tomato^+^ compartment. **c.** qRT-PCR of Tomato expression in CD45^−^/Tomato^+^ sorted tumor cells obtained from tumors originated in transplanted Tomato/neu animals, relative to constitutively expressing Tomato/CRE cells. The positive control is RNA extracted from a constitutively expressing Tomato strain (tomato/cre). Negative control is RNA extracted from the spleen of a LoxP-tdTomato animal (spleen loxP). Tomato expression is present only in Tomato^+^ sorted cells from primary tumors of selected transplanted mice (indicated with the bmt code). Samples were run in triplicates and normalized on β-actin mRNA. Data are represented as mean ±S.D. (d-f) Representative images of Tomato staining on FFPE sections of different tumors with the highest Tomato positive cell content (d and e: 10x, f: 20x, scale bars = 50 μm). In many cases the cells seem to be clustered, suggesting a clonal derivation from single fused cells.

Fifty-eight % of the tumors analyzed (26/45) showed the presence of Tomato^+^ cells restricted to the CD45^−^ compartment. Interestingly, we observed both Tomato^+^ and Tomato^−^ tumors grown in the same animal. The percentage of Tomato^+^ cells (mean = 0.86%, S.E.M. = ±0.66%) was lower compared to the GFP/RFP model (Figure 5a), with the exception of the tumor of one mouse, which showed very high levels of Tomato cells (17.4%).

Figure 5b shows the relative MFI (RFI) of ErbB2 and F4/80 respectively in the Tomato^+^ populations of the CD45^−^ compartment, showing that these markers are expressed in 50% of Tomato^+^ tumors (13/26). In this second chimeric model, the number of Tomato^+^ cells in the CD45^+^ compartment was too low to be further analyzed for the RFI of ErbB2 and F4/80.

Finally, qPCR analysis of Tomato expression performed on Tomato^+^ sorted cells from 6 tumors further confirmed the expression of the gene (Figure 5c).

Histological analysis with a Tomato specific antibody was performed on tumors with the highest Tomato positive cell content. Interestingly, positive cells were easily detectable and were clustered, in keeping with their clonal origin following fusion events (Figures 5d-5f). Immunohistochemistry analysis of lung metastasis again did not show any Tomato cells in all the metastases examined.

**Figure 6:**
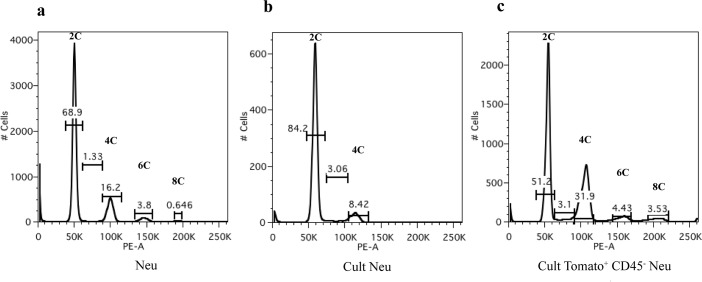
DNA content analysis by flow cytometry Propidium iodide (PI) staining and flow cytometric analysis of **a.** neu tumor cells derived from a MMTV-neu mouse and immediately fixed, **b.** cultured neu tumor cells and **c.** cultured sorted Tomato^+^ CD45^−^ tumor cells. The x-axis shows the DNA content revealed by PI fluorescence and the y-axis displays the number of cells analyzed. Percentages of cells in each population, based on DNA content, are indicated on the peaks.

Unfortunately, both in the first and in the second chimeric model, we failed in *in vitro* culturing sorted GFP^+^/RFP^+^ and Tomato^+^ cells respectively to better characterize these populations. The difficulty in culturing the fused colored cells might be related to a low viability of aneuploid cells during *in vitro* culture [25, 26]. In an effort to better clarify this aspect we performed DNA content analysis on tumor cells through a flow cytometry approach (Figure 6). As expected, the suspension of neu tumor cells derived from a MMTV-neu mouse and immediately fixed for the analysis showed a dominant diploid population (2C that corresponds to G1 diploid cells) together with a 4C peak containing both G2/M diploid cells and G1 tetraploid cells (4N) and two minor peaks 6C and 8C of hyperdiploid cells (Figure 6a). Interestingly, this hyperdiploid population decreased during cell culture, since only a diploid population containing an increased number of S cells (from 1.33% to 3.06%) and a lowest 4C peak (from 16.2% to 8.42%) was evident (Figure 6b). The analysis of sorted Tomato^+^ CD45^−^ tumor cells cultured for few passages showed an enrichment of hyperdiploid cells with the increase of the 4C peak (more than 20% of 4C cells) which includes tetraploid cells, and the presence of two additional peaks of hyperdiploid cells (Figure 6c). The presence of these G1 tetraploid cells is confirmed by a larger 4C peak compared to the cultured neu cells, despite the same percentage of S cells. This pattern is compatible with our hypothesis of fused cells.

Taken together, the two chimeric models analyzed gave consistent results, showing fusion between neoplastic and non neoplastic cells with features of macrophagic cells. The clear histological images obtained with the Tomato model demonstrate that these cells are not phagocytic cells but alive proliferating cells.

## DISCUSSION

The role of the microenvironment surrounding neoplasias has recently been added to the list of the factors playing a role in tumor progression [27]. In this regard, many studies have pointed to the macrophage as the most important cell representing an effective innate immune barrier to tumor growth, although other findings suggest that it can also play a role in promoting tumor invasion and dissemination (reviewed in [28] [29]). Tumor associated macrophages (TAM) could exert their pro-tumoral effects by suppressing other immune cells, by secreting angiogenic factors, by modifying the matrix composition or even by contributing to the gene pool of the neoplastic cell by cell fusion [30]. In the latter case, this could be instrumental in providing the neoplastic cell with a set of already activated genes devoted to motility and migration, which could be exploited to invade local tissues and disseminate to distant organs.

Although this concept is not novel [31], having been proposed more than a century ago, *in vivo* data supporting this hypothesis are still lacking. So far, to our knowledge, the best approach used has been a parabiosis model which allows the transfer of GFP^+^ donor hematopoietic normal cells into a spontaneously arising intestinal neoplasia positive for beta-gal [32]. However, this model was not implemented to evaluate the acquisition of metastatic potential.

The results reported here confirm that tumors spontaneously arising *in vivo* contain a measurable amount of cell fusion events and identify the macrophage as one of the most relevant partner, in agreement with previous studies. The fact that similar data were obtained with two different strategies further supports the conclusion. The lower levels detected with the BMT approach might be due to the fact that the detection system interrogates only hematopoietic derived cells. In this regard, it is possible that other normal cells, such as endothelial or stromal ones, could also be involved in fusion with neoplastic cells. Of note, it is also well-documented in literature that, with the exception of microglia, most tissue-resident macrophages derive from donor bone-marrow hematopoietic precursors after lethal irradiation and bone marrow transplantation [33–35]. Therefore in the second (Tomato) approach only macrophages derived from the CRE donor are present in the bone marrow chimeras, so they could not fuse with the non activated-Tomato positive macrophages, most of which have been eliminated. Since the FACS analysis of the GFP/RFP model has suggested that most CD45^+^ fused cells are probably the result of intra-hematopoietic cell fusion, this could explain why fused CD45^+^ cells are absent in the Tomato model.

The co-expression of ErbB2 and F4/80 receptors in fused cells only in the CD45^−^ compartment can be ascribed to the nuclear reprogramming following the collision between two different expression programs, as already reported by Powell and co-workers [32]. In their work, as we found in our study, they have demonstrated the expression of the macrophage-specific *F4/80* gene in cell fusion hybrids, but only until four weeks post-transplantation. Nonetheless, in contrast with their work, in our study the F4/80 protein was detectable, even if with different expression levels in the BMT chimeras of the second approach, at the day of sacrifice of the animals, at around 6 months after bone marrow transplantation. The F4/80 receptor was found to be necessary for the induction of efferent CD8+ regulatory T cells responsible for peripheral immune tolerance [36] and this might explain the presence of this receptor in fused cells as a defense strategy to circumvent immune system. Concerning the lack of CD45 expression in fused cells, we confirmed the results of a previous study [37] in which intestinal cell fusion hybrids between transplanted bone marrow-derived cells and both normal and neoplastic intestinal epithelium in BMT chimeras lacked CD45 expression, showing that nuclei of fused cells have been reprogrammed and no longer express the hematopoietic marker.

An important aspect of our experimental plan is that it exploits an *in vivo* system to solve the problem of whether the fusion between normal and neoplastic cells can confer a further level of malignancy to the neoplastic cell. The MMTV-neu mouse develops lung metastases with high frequency and high repeatability providing a simple but clear-cut system to show the role of fused cells. We reasoned that, if cell fusion is relevant to tumor progression, a number of lung metastases should display both markers, either completely, if the advantage was in the acquisition of dissemination ability, or partially, if the cell fusion occurred within the lung during the colonization phase. We indeed confirmed that fusion between neoplastic and normal cells, including macrophages, did occur. However, the fact that we were unable to detect fused cells in any metastases examined strongly suggests that macrophage fusion does not play a role in the metastatic phenotype. The two chimeric models were chosen to clearly detect fused cells in lung metastases by immunohistochemistry assuming that the metastatic growth was clonally derived from a single or few “colored” tumor cells; however, if the metastatic foci were polyclonal, only a portion of cells of the foci would be fused and FACS analysis of lung metastasis would be necessary.

In spite of its numerous similarities between our model and human breast cancer [19], the strategy chosen here has several limitations. Perhaps the most relevant is the extreme aggressive behaviour of mammary and salivary tumors driven by the *neu* oncogene which is expressed at very high levels from the MMTV-LTR promoter. It could be possible that this intrinsic feature, which is shared only by a minority of human breast cancers, makes the neoplastic cells so well equipped with invasive genes that they do not need any additional help from macrophage fusion in order to disseminate. Moreover, cell fusion could be more relevant in tumors with a large inflammatory component [38] which, although present in the MMTV-neu model, is not extensive.

An additional point raised recently was the possibility that transfer of cytosolic material could occur *via* extravescicular moieties [39]. Zomer and collegues has used a CRE-based reporter system similar to that used in our study, showing activation of the Tomato gene in the recipient cell, likely due to mRNA transfer coding for the CRE recombinase. Although we cannot formally exclude this possibility, the presence of both ErbB2 and F4/80 markers on Tomato positive cells suggests that massive fusion, with cooption of the complete genome, has occurred. In addition, although in the CRE-Tomato approach the activation of the Tomato reporter could be accomplished even by a short exposure to the CRE recombinase protein and thus being compatible with an mRNA transfer *via* extravescicular moieties, the results obtained with the GFP/RFP strategy would require continuous expression of both reporters in the same cell, which in turn would necessitate continuous transfer of such mRNAs.

In summary, we showed that macrophages fuse with neoplastic cells in our model, thus confirming a large amount of data in more artificial models. However, in our opinion, the nature of these events, whether they end in apoptosis, whether simply represent a reaction by the innate immune system against tumor cells or whether they can provide an additional path to malignancy is not yet clarified. Our data on metastasis distribution in a spontaneously arising tumor model are against a functional relevance of cell fusion, but other models with different pathogenesis are worth to be further investigated.

## MATERIALS AND METHODS

### Animals

The mouse facility was maintained at a temperature of 23°C (± 0.5°C). The light cycle was set at 14/10 h (light/dark). Animals were given ad libitum access to food and water. The experimental procedures were carried out in agreement with Italian regulations (D.Lgs. 116/92) and EU guidelines (2010/63/EU).

EGFP [40] and mRFP1 transgenic mice were purchased from The Jackson Laboratory (stock number 003291 and 005884 respectively) (Bar Harbor, ME). The EGFP transgenic mice (hereafter referred to as “GFP”) were mated with a CD1 background. The mRFP1 transgenic mice (hereafter referred to as “RFP”) were mated with a C57BL/6N background. Both GFP and RFP mice were then mated with MMTV-neu transgenic mice (hereafter referred to as “neu”) which we constructed several years ago [20]. This strain, in a CD1 background, develops breast tumors in 100% of the females within about 5 months of age and salivary gland tumors in males [19]. Since starting from the appearance of the first tumors in the MMTV-neu animals, the development of the pathology in some mice exhibits an unpredictable evolution. With the exception of an animal which was killed at two months, all the chimeras were sacrificed between 4 and 7 months of age, depending on the timing of achievement of the maximum tumor diameter allowed by the animal welfare guidelines for the correct management of this transgenic line or whenever an animal showed signals of suffering. This end point is in agreement with the previous work [19], showing that at about 5 months of age MMTV-neu mice develop lung metastases.

CMV-CRE and LoxP-tdTomato transgenic mice carrying the Cre recombinase gene under the control of a human CMV promoter or the tandem dimer Tomato gene (tdTomato), a red fluorescent protein variant, were purchased from The Jackson Laboratory (stock number 006054 and 007905, respectively). In the LoxP-tdTomato transgenic mice (hereafter referred to as “Tomato”) a *loxP*-flanked STOP cassette prevents transcription of the downstream Tomato protein, which is activated when the mouse is crossed to the CMV-CRE mouse (hereafter referred to as “CRE”).

### Generation of chimeric mice by morula aggregation

Chimeric mice were produced by the aggregation of two morula stage embryos that were obtained by mating GFP/neu females with GFP/neu males and RFP/neu females with RFP/neu males, following standard techniques [41]. The chimeric offsprings were identified based on the fluorescence of EGFP and mRFP1. DNA obtained from the tails of the chimeric mice was analyzed by PCR for the presence of the neu transgene with appropriate primers (R2-ERBB2-F: CTGCAGGAAACTGAGTTA, R2-ERBB2-R: CTCTCAACACCTTGATAG; program: 95°C for 2 min; 95°C for 30 sec, 57°C for 30 sec, 72°C for 1 min, 35 cycles; 72°C for 5 min).

### Generation of bone marrow chimeras

LoxP-tdTomato females were mated with MMTV-neu transgenic male mice. Six-eight weeks old double transgenic MMTV-neu/LoxP-tdTomato animals (Tomato/neu) were lethally irradiated with a dose of 900 cGy. Two hours later, mice were injected in the retro-orbital plexus with 4*10^6^ whole bone marrow (BM) cells obtained by flushing femurs harvested from sex-mismatched CRE animals. Recipient animals were treated with Gentamicin (0.8 mg/ml in the drinking water) from 10 days before to 14 days after irradiation.

BM engraftment was assessed by means of Quantitative PCR on genomic DNA extracted from peripheral blood adapting the protocol described in [42]. Genomic DNA (gDNA) was extracted using QIAamp DNA Blood mini kit (Qiagen, Germany) from peripheral blood of transplanted animals at the day of sacrifice and Cre and Transferrin Receptor (Tfrc) specific probes designed for TaqMan copy number assay (Cat. # 4400291 and Cat. # 4458366) (both from Life Technologies, Milan, Italy) were used. A reference standard curve was made with 8 serial dilutions ranging from 100% CRE gDNA to 0.1% CRE gDNA diluted in Tomato/neu gDNA in a final constant concentration of 5 ng/μl. As negative control, gDNA from Tomato/neu animals was used. Ten ng of gDNA obtained from transplanted animals and from standard curve serial dilutions were loaded and qPCR was performed using a ViiA7 Real-Time PCR system (Life Technologies) with the following program: Hold Stage, 95°C for 10 minutes for 1 cycle; PCR Stage, 95°C for 15 seconds and 60°C for 1 minute for 40 cycles. Amplification efficiency was evaluated and Ct values of Cre probe were normalized on the Ct values of the Tfrc gene and then quantified on the normalized standard curve by using the 2^−ΔΔCT^ method as described in [43]. Each sample was run in triplicate on at least 2 independent runs.

### Quantitative real-time PCR

Total RNA was extracted from sorted Tomato positive and Tomato negative cells with RNeasy Plus Micro kit (Qiagen) and cDNA was synthetized using High Capacity cDNA reverse transcription kit (Life Technologies). Quantitative real-time PCR was performed on a Viaa7 system (Life Technologies) by Sybr Green chemistry (Life Technologies). Each sample was analyzed in triplicate and the level of expression relative to constitutively expressing Tomato/CRE cells was determined by normalization to beta-actin (Actb) mRNA. Relative fold change expression was computed using the 2^−ΔΔCT^ method as described in [43]. The primers used were: TdTomato forward: GGCATTAAAGCAGCGTATCC; TdTomato reverse: CTGTTCCTGTACGGCATGG; ActB forward: CTAAGGCCAACCGTGAAAAG; ActB reverse: ACCAGAGGCATACAGGGACA.

### FACS sorting and analysis

Tumor cell suspensions were obtained by manual smashing followed by enzymatic digestion (Collagenase 0.01 mg/ml and DNAse-I 0.05 mg/ml) of a representative fraction of the total tumor explanted. Cell surface staining of one million single cell suspension, obtained upon filtration with 70 μm cell strainers, were performed in staining buffer (PBS plus 2% Fetal Calf Serum) for 30 minutes, on ice, in the dark, with the appropriate combinations of saturating concentrations of the following conjugated monoclonal antibodies (mAbs) obtained from either BD Biosciences (Milan, Italy), eBioscience (Hatfield, UK), or BioLegend (London, UK): CD45 (30-F11), F4/80 (BM8), CD11b (M1/70). Anti-c-ErbB2/c-Neu (OP16 or Ab4 clone 7.16.4) antibody was purchased from Merck-Millipore (Vimodrone, Italy) and its isotype control IgG2a from Invitrogen (Life Technologies); both antibodies were labeled with Alexa Fluor^®^ 647 using Zenon labelling kit (Molecular Probes, Life Technologies) following the manufacturer's instructions. A pre-incubation with anti-CD16/CD32 (clone 93 from eBioscience) was performed in order to minimize aspecific interaction of the antibodies with Fc receptor expressed on the cell surface. Dead cells were excluded using LIVE/DEAD Fixable Cell Stain Kit (Life Technologies). Cell to cell aggregates were excluded from the analysis plotting either morphological parameters SSC-Area and FSC-Area *versus* SSC-Width and FSC-Width respectively. Stained cells were analyzed using LSR Fortessa (4 lasers) flow cytometer (BD Biosciences). Cell sorting experiments were performed using a FACSAria III (4 lasers; BD Biosciences). FACS Diva software (BD Pharmingen, BD Biosciences) and Flow-jo (Tree Star, Ashland, OR, USA) were used for data acquisition and analysis respectively.

### Histochemistry and Immunofluorescence

A representative fraction of each tumor and whole lungs from all the animals described were fixed in 4% formalin and then paraffin embedded (FFPE) using standard protocols. For Tomato detection in tumors and lungs, 3 μm slices were stained with Anti-RFP antibody ab124754 (Abcam, Cambridge, UK) diluted 1:400 in PBS containing 0.05% Tween 20. Revealing was performed using Mach 1 HRP-polymer (Biocare Medical, Concord, CA, USA) incubation followed by the revelation with Betazoid DAB (Biocare Medical). Negative controls were conducted by omitting the primary antibody. Nuclei were counterstained with Mayer's hematoxylin solution (Dako, Milan, Italy).

Double immunofluorescence staining was performed on FFPE 3 μm slices of tumors and lungs. We used the Anti-GFP, rabbit IgG fraction, biotin-XX conjugated monoclonal antibody (Life Technologies) diluted 1:200, and the Anti-RFP antibody ab124754 (Abcam) diluted 1:400. Secondary antibodies were Streptavidin Alexa Fluor^®^ 488-conjugated (1:500) and Alexa Fluor^®^ 594-conjugated donkey anti rabbit (1:500) respectively (Life Technologies). Nuclei were counterstained with DAPI (10 μg/ml). Sections were mounted with the antifade medium FluorPreserve Reagent (Calbiochem, Merck-Millipore) and analyzed with an Olympus Fluoview FV1000/TIRF laser scanning confocal microscope.

### Lung metastasis analysis

Lungs harvested from all animals were formalin fixed and paraffin embedded and systematically sectioned through the entire length collecting at least five 3 μm sections every 100 μm. Presence and number of metastasis were observed on the first slide of each section after Hematoxylin and Eosin (H&E) staining. If metastasis count scored positive, the next slice was used for immunofluorescence or immunohistochemistry analysis as described in the previous section.

### Tumor cell culture and DNA content analysis by flow cytometry

Tumor-derived single cell suspensions were cultured in RPMI supplemented with 20% fetal bovine serum (FBS), 12 mM HEPES buffer, 2 mM L-glutamine, 10 μM non-essential amino acids, 1 mM sodium pyruvate, 100 μg/ml streptomycin and 100 U/ml penicillin. One million of fresh neu tumor cells, cultured neu tumor cells and cultured sorted Tomato^+^ CD45^−^ tumor cells were fixed in 70% ethanol, stored for at least 2 h at 4°C and stained with PI/RNase staining buffer (BD Pharmingen). Cells were then analyzed by FACSCanto II (BD Biosciences), using Flow-jo software.

## SUPPLEMENTARY MATERIALS FIGURE


