# Supplementary material for: Fusion between cancer cells and macrophages occurs in a murine model of spontaneous neu+ breast cancer without increasing its metastatic potential
Source: Oncotarget. 2016 Aug 22;7(38):60793–806. doi: 10.18632/oncotarget.11508 (PMC5308617; doi:10.18632/oncotarget.11508)
Supplement: Supplementary file 1 [file oncotarget-07-60793-s001.pdf]

Fusion between cancer cells and macrophages occurs in a murine model of spontaneous *neu*$^{+}$ breast cancer without increasing its metastatic potential

Supplementary Material

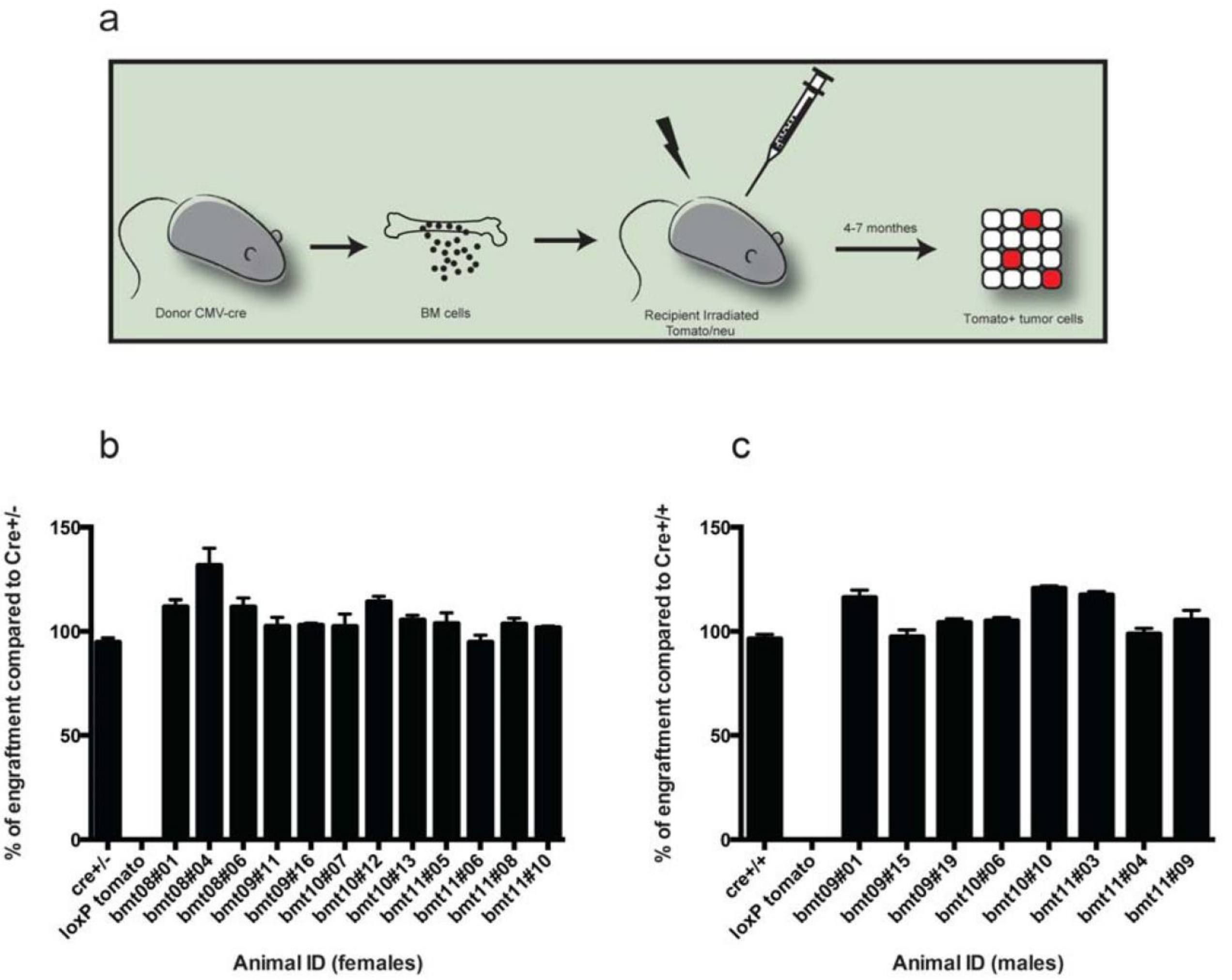

Supplementary Fig. 1. Bone Marrow Transplant (BMT) chimeric model. (**a**) Outline of the BMT chimeric model. Male or female Tomato/neu animals were lethally irradiated and subjected to BMT from female CRE$^{+/+}$ or male CRE$^{+/-}$ animals, respectively. Fusion between tumor cells and hematopoietic-derived cells would induce expression of Tomato fluorescent protein. (**b** and **c**) Chimerism assessment in the BMT model by qPCR. All the transplanted animals showed high levels of bone marrow chimerism ranging from 95% to 100% as revealed by qPCR on peripheral

blood derived genomic DNA. (**b**) Percentage of engraftment in female Tomato/neu animals transplanted with bone marrow from male $CRE^{+/-}$ animals. (**c**) Percentage of engraftment in male Tomato/neu animals transplanted with bone marrow from female $CRE^{+/+}$ animals (the *CRE* transgene is located on the X chromosome).
